# Evaluation of a Novel High-Definition PCR Multiplex Assay for Simultaneous Detection of Tick-Borne Pathogens in Human Clinical Specimens

**DOI:** 10.1128/JCM.01655-19

**Published:** 2020-02-24

**Authors:** Salika M. Shakir, Christopher R. Mansfield, Elizabeth D. Hays, Marc Roger Couturier, David R. Hillyard

**Affiliations:** a Department of Pathology, University of Utah School of Medicine, Salt Lake City, Utah, USA; b ARUP Institute for Clinical and Experimental Pathology, Salt Lake City, Utah, USA; Mayo Clinic

**Keywords:** high-definition PCR, tick-borne pathogen, multiplex panel

## Abstract

The incidence of tick-borne infections in the United States has risen significantly in the past decade. Ticks can transmit a variety of pathogens, including bacteria, protozoa, and viruses, that can cause serious illnesses. Therefore, the use of rapid, sensitive, and specific multiplex tests is important to identify the pathogen(s) in the acute phase and determine appropriate treatment to minimize the severity of the disease. The purpose of this study was to evaluate ChromaCode’s research use only (RUO) nine-target high-definition PCR (HDPCR) tick-borne pathogen (TBP) panel using 379 retrospective, remnant whole-blood and synovial fluid specimens previously submitted to Associated Regional and University Pathologists (ARUP) Laboratories and tested by clinically validated real-time PCR assays for Ehrlichia spp., Anaplasma phagocytophilum, Babesia spp., or Lyme Borrelia spp. The performance characteristics evaluated included positive percent agreement (PPA) and negative percent agreement (NPA) with the ARUP laboratory-developed tests (LDTs). All tested targets had an initial PPA greater than 97.0%, except Ehrlichia ewingii, with a PPA of 88.9%. The NPAs for all targets were between 98.8% and 100%. The TBP panel detected three coinfections, with two of Babesia microti and A. phagocytophilum and one of B. microti and *E. chaffeensis*, which were confirmed by the LDTs. There were 16 samples with discordant results compared to the LDT results, five of which were resolved by repeat testing on the TBP panel and bidirectional sequencing. Following discrepant resolution, the final PPA and NPA for the TBP panel were 97.7% (95% confidence interval [CI], 95.2% to 99.0%) and 99.6% (95% CI, 99.3% to 99.8%), respectively, with an overall agreement of 99.5% (95% CI, 99.2% to 99.7%) with the LDTs.

## Abstract (precis)

The incidence of tick-borne infections in the United States has risen significantly in the past decade. Ticks can transmit a variety of pathogens, including bacteria, protozoa, and viruses, that can cause serious illnesses. Therefore, the use of rapid, sensitive, and specific multiplex tests is important to identify the pathogen(s) in the acute phase and determine appropriate treatment to minimize the severity of the disease.

## INTRODUCTION

Tick-borne illnesses, including Lyme disease, human granulocytic anaplasmosis, babesiosis, human monocytic ehrlichiosis, and relapsing fever, are the most common tick-borne diseases in the United States and have continued to rise over the last decade (1–3). Clinical presentations of tick-borne infections can range from mild to life-threatening, with symptoms including fever, headaches, myalgia, arthralgia, nausea, and vomiting, often overlapping in the early stages of disease. Most tick-borne pathogens are difficult to culture in the laboratory; thus, diagnosis has been based primarily on clinical presentation, history of exposure in areas of endemicity, microscopic examination of blood smears, and serological tests (https://www.cdc.gov/ticks/tickbornediseases/index.html) (1, 4). Though serologic testing may support laboratory evidence of tick-borne disease, it is limited by decreased sensitivity in the acute phases of disease and poor clinical specificity (4, 5). Nucleic acid amplification tests (NAAT) offer the advantages of directly detecting these pathogens during early infection. Real-time PCR tests for tick-borne diseases are available through the Centers of Disease Control and Prevention (CDC), state health laboratories, and certain reference laboratories, but these vary in sensitivity and specificity and are limited to singleplex assays or those that detect three or four targets only (6–10). This highlights an unmet need for a multiplex syndromic panel for accurate identification of these tick-borne disease agents. A comprehensive multiplex panel that targets a broader array of tick-borne pathogens will be necessary for the early detection and effective management of disease.

The purpose of this study was to evaluate ChromaCode’s research use only (RUO) high-definition PCR (HDPCR) tick-borne pathogen (TBP) panel (Carlsbad, CA) using whole-blood and synovial fluid specimens compared to the Associated Regional and University Pathologists (ARUP) laboratory-developed tests (LDTs) currently used for clinical testing. The TBP panel is a multiplex, 4-color channel PCR assay which allows for the simultaneous detection of nine tick-borne pathogens in a single well by endpoint signal intensity. The TBP panel detects Anaplasma phagocytophilum, Ehrlichia chaffeensis, Ehrlichia ewingii, Ehrlichia muris eauclarensis, Borrelia miyamotoi, *Borrelia* group 1 (B. burgdorferi and B. mayonii), *Borrelia* group 2 (B. hermsii, B. parkeri, and B. turicatae), Babesia microti, and Rickettsia spp. A recent study by Buchan et al. describes a preliminary evaluation of the TBP panel for the identification of tick-borne pathogens in human clinical and simulated specimens (11). The study findings describe high specificity (>98%) and sensitivity (100%) for A. phagocytophilum, B. miyamotoi, and *Rickettsia* spp. among clinical specimens, in addition to 100% analytical sensitivity for all targets and a combined analytical specificity of 99.5% in simulated samples. The conclusions of this study focused on the potential utility and clinical impact of implementing the TBP panel; however, because it was a prospective study, a minimal number of positive clinical samples were evaluated. For a broader understanding of the performance of the assay, we tested a large set of well-characterized, clinical specimens archived at ARUP Laboratories that were positive for six of the nine targets in the TBP panel. Our retrospective study design evaluated the TBP panel to detect tick-borne pathogens of low incidence in a standard quantitative PCR (qPCR) instrument and compared the performance characteristics to those of LDTs. The results of this study demonstrate the potential value of the TBP panel in detecting common tick-borne pathogens in a simple, high-throughput, scalable assay that may be easily adopted in clinical laboratories.

## MATERIALS AND METHODS

### Clinical samples.

A total of 371 retrospective, whole-blood samples that were archived at ARUP Laboratories and previously tested via laboratory-developed PCR tests for the detection of *Ehrlichia* spp., Anaplasma phagocytophilum, *Babesia* spp., and Lyme *Borrelia* spp. were enrolled in this study. Eight synovial fluid samples were included to evaluate the analytical performance of the Lyme *Borrelia* sp. target in the TBP panel. Specimens were deidentified under a study protocol approved by the University of Utah institutional review board (IRB; protocol 00042995). The results of the reference method were blinded prior to testing with the TBP panel.

### DNA extraction.

Nucleic acids were extracted from 200 μl of whole blood or synovial fluid using the chemagic magnetic separation module (MSM) I automated extraction platform (PerkinElmer, Waltham, MA), according to standard laboratory procedures. Ten microliters of internal control provided by ChromaCode was added to each of the samples prior to extraction at a concentration of approximately 10^3^ copies/reaction. The internal control served as a control for both extraction efficiency and presence of PCR inhibitors. The samples were eluted in 50 μl of elution buffer.

### Instrument characterization.

All testing for this study was performed at ARUP Laboratories on a QuantStudio 12K Flex system (Thermo Fisher Scientific, Waltham, MA) using the fast 96-well block. Prior to TBP testing, an instrument characterization step was performed to equalize the instrument-specific noise profile on the QuantStudio 12K system using synthetic DNA provided in the TBP panel equalization kit, according to the manufacturer’s instructions for use (IFU). Briefly, four individual MicroAmp fast optical 96-well reaction plates of synthetic DNA template corresponding to the four individual fluorophore channels at known concentrations were mixed with HDPCR master mix in every well of a 96-well plate and run according to the manufacturer’s IFU. The results from each of these four runs were uploaded into ChromaCode Cloud, and a noise correction mask specific to the QuantStudio 12K instrument used in the study was generated by ChromaCode’s proprietary signal processing software analysis.

### TBP panel design and testing.

The TBP panel is a single-well, 4-channel assay that detects nine common tick-borne pathogens and also includes an internal control. The TBP panel has the following design: 6-carboxyfluorescein (FAM) channel, *Borrelia* group 1 (B. burgdorferi and *B. mayonii*), Ehrlichia chaffeensis, and Borrelia miyamotoi; ATTO532 channel, *Rickettsia* spp., Ehrlichia muris *eauclarensis*, and Anaplasma phagocytophilum; ROX channel, internal control; and ATT0647N channel, *Borrelia* group 2 (B. hermsii, *B. parkeri*, and *B. turicatae*), Babesia microti, and Ehrlichia ewingii. The specific genes targeted by the TBP panel are described by Buchan et al. (11). The TBP assay thermocycling parameters were as described in the manufacturer’s IFU, as follows: stage 1, initial denaturation for 1 min at 95°C; and stage 2, denaturation for 10 s 95°C and annealing for 60.0°C for 2 min for 65 cycles.

For the TBP testing in the study, 5 μl of extracted DNA from whole blood or synovial fluid was added to 15 μl of master mix containing primers, probes, and enzyme (all provided in TBP test kit) in 96-well fast plate. Four-plate calibrators provided in the TBP test kit run with each plate to set the levels for target classification. The results for each TBP test were analyzed in ChromaCode Cloud by uploading the raw data file (.xls file) from the study instrument to the study account in ChromaCode Cloud. A report of positive for a target, negative, or invalid result for each sample is generated. Positive percent agreement (PPA) and negative percent (NPA) agreement compared with the ARUP LDTs were calculated.

### ARUP Laboratories real-time PCR assays for tick-borne pathogens.

The comparator methods for the study were ARUP’s real-time PCR LDTs for *Ehrlichia* spp. and A. phagocytophilum, *Babesia* sp., and Lyme *Borrelia* sp. performed on the QuantStudio 12K Flex instrument (Thermo Fisher Scientific, Waltham, MA). The assay for *Ehrlichia* and *Anaplasma* spp. detects *E. chaffeensis*, *E. muris*-like pathogen, *E. ewingii*, and Ehrlichia canis (without differentiating *E. ewingii* and *E. canis*), as described by Harris et al. (7). The *Babesia* assay amplifies a 190-bp segment of the 18S rRNA of *Babesia* spp. with a probe specific for B. microti and a probe to detect other *Babesia* spp. (B. duncani, B. divergens, *Babesia* sp. strain MO-1, and *Babesia* sp. strain EU1), as described by Couturier et al. (12). For the Lyme *Borrelia* assay, primers and probes designed to amplify a 68-bp segment of the *ospA* gene were used. The primer sequences were GA*AAAAATATTTATTGGGA*ATAGGTCT for BOR-L3 and GGCTGCTAACATTTTGCTTACAT for BOR-E3, and the *Borrelia* probe sequence BOR-FAM1 was MGB-FAM-G*AGCCTTA*A*TA*GCA*TG-EDQ (G*indicates super-G modified base, A* indicates super-A modified base; MGB, minor groove binder; FAM, 6-carboxyfluorescein; and EDQ, Eclipse dark quencher [ELITech Group, Bothell, WA, USA]). The reaction was prepared by using a 5× GoTaq probe qPCR master mix and 4.5 mmol/liter MgCl_2_ (Promega, Madison, WI, USA) with the following amplification parameters: 50.0°C for 10 min, denaturation at 95.0°C for 2 min, and 50 cycles of 95.0°C for 5 s, 56.0°C for 20 s, and 76.0°C for 20 s. The *ospA* gene is conserved among the Lyme *Borrelia* species and can also detect Borrelia afzelii and Borrelia garinii.

### Discrepant analysis.

Samples with discrepant results initially underwent repeat testing on the TBP panel. Only dual-positive samples that repeated as dual positive with the TBP panel were tested on the LDT for *Ehrlichia* spp., *Anaplasma* spp., and *Babesia* spp. to determine whether the TBP panel detected a coinfection not originally detected by the LDT. The final call for discordant samples was made based on the results of a repeat TBP panel result and repeat LDT result. Those samples that could not be resolved by these two methods were further tested by PCR and bidirectional sequencing.

### Discrepant resolution by PCR and bidirectional sequencing.

Discrepant sample resolution was executed by PCR and bidirectional sequencing. The primer sequences used for amplification and bidirectional sequencing are proprietary and not included in the manuscript. Samples were amplified using AmpliTaq Gold 360 DNA polymerase (catalog no. 4l398823; Applied Biosystems). The amplification was performed for 40 cycles with initial denaturation for 10 min at 95°C, denaturation for 30 s at 95°C, annealing for 30 s at 50°C, extension for 1 min at 72°C, and a final extension for 7 min at 72°C. The PCR was performed using the Bio-Rad T100 thermal cycler. The amplification products were analyzed by 2% agarose gel electrophoresis, and DNA was sequenced by the Sanger method at Retrogen, Inc. (San Diego, CA). Sequencing analysis was performed using the KB Basecaller algorithm with a Phred Q20 score.

## RESULTS

**FIG 1:**
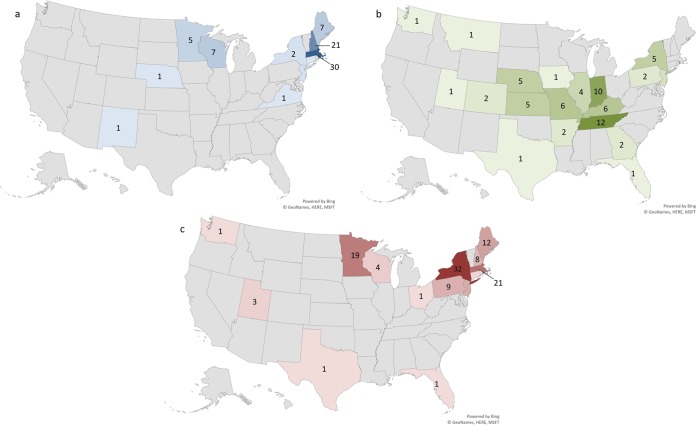
(a to c) Map of the number of positive cases of A. phagocytophilum (a), *E. chaffeensis* (b), and B. microti (c) in the United States sent to ARUP laboratories for reference testing.

A total of 371 archived whole-blood samples and eight synovial fluid clinical samples that were submitted to ARUP Laboratories for PCR between 2014 and 2018 for the detection of *Ehrlichia* spp., *Anaplasma* spp., *Babesia* spp., or *Borrelia* spp. were tested using the TBP panel. These samples included 325 samples positive by PCR for any of *E. chaffeensis*, *E. ewingii*, *E. muris*-like, A. phagocytophilum, B. microti, or Lyme *Borrelia* spp. Fifty-three negative whole-blood samples were also included. Figure 1 shows the distribution of positive specimens included in the study across various U.S. states. The case incidence correlates with the areas where cases of A. phagocytophilum, *E. chaffeensis*, and B. microti have been previously reported (13). However, these may not necessarily be the state where the patient was infected. The majority of the A. phagocytophilum-positive samples tested (*n* = 78) were from Massachusetts (38%) and New Hampshire (27%), followed by Maine and Wisconsin (9%) (Fig. 1a). The cases of positive *E. chaffeensis* samples (*n* = 70) were distributed across 20 states, including Tennessee (17%), Indiana (14%), Missouri, and Kentucky (8.5%) (Fig. 1b). The majority of B. microti-positive samples (*n* = 124) were from New York (26%), Massachusetts (17%), Minnesota (15%), Maine (10%), and New Jersey (8%) (Fig. 1c).

Table 1 shows the initial performance of the TBP panel in comparison to LDTs. The TBP panel call rate was 99.7% (378/379). One sample was excluded from the overall analysis due to an internal control failure causing an invalid result. All tested targets had a positive percent agreement (PPA) greater than 97.0%, except for *E. ewingii* (88.9%). All eight synovial fluid specimens tested positive for *Borrelia* group 1 (PPA, 100%; 95% confidence interval [CI], 59.8% to 100%). The PPA values for A. phagocytophilum, B. microti, and *E. chaffeensis* were 98.8% (95% CI, 92.6 to 99.9%), 97.7% (95% CI, 92.8 to 99.4%), and 97.4% (95% CI, 90.2 to 99.6%), respectively. None of the samples tested were positive for spotted fever *Rickettsia* spp., *Borrelia* group 2 (relapsing fever *Borrelia*), or B. miyamotoi. The negative percent agreement (NPA) values for all targets were between 99.3% and 100%, except for B. microti (98.8%) and *Borrelia* group 2 (98.9%). The total PPA and NPA values for the HDPCR TBP panel were 97.7% (301/308) and 99.5% (3,082/3,095), respectively, with an overall accuracy of 99.4% (95% CI, 99.1% to 99.6%) compared to the LDTs.

**TABLE 1:** Performance of the TBP RUO assay using whole-blood or synovial fluid clinical samples (*n* = 378)^*a*^

Analyte	Positive percent agreement^*b*^			Negative percent agreement		
	TP/(TP + FN)	%	95% CI	TN/(TN + FP)	%	95% CI
Anaplasma phagocytophilum	83/84	98.8	92.6–99.9	292/294	99.3	97.3–99.9
Babesia microti	125/128	97.7	92.8–99.4	247/250	98.8	96.2–99.7
Borrelia miyamotoi	0	0	0	378/378	100	98.8–100
*Borrelia* group 1	8/8	100	59.8–100	368/370	99.5	97.9–99.9
*Borrelia* group 2	0	0	0	374/378	98.9	97.1–99.7
Ehrlichia chaffeensis	76/78	97.4	90.2–99.6	299/301	99.4	97.6–99.9
Ehrlichia ewingii	8/9	88.9	50.7–99.4	369/369	100	98.7–100
Ehrlichia muris *eauclarensis*	1/1	100	5.5–100	377/377	100	98.7–100
*Rickettsia* spp.	0	0	0	378/378	100	98.8–100
Total	301/308	97.7	95.3–99.0	3,082/3,095	99.5	99.3–99.8

a Overall percent agreement was 99.4% (95% CI, 99.1% to 99.6%) compared to the ARUP laboratory-developed assay results.

b No samples were positive for B. miyamotoi, *Borrelia* group 2, and *Rickettsia* spp.

There were 16 samples with 20 discrepant results compared to the LDTs for tick-borne infections in the initial analysis (Table 2). All 16 samples were retested on the TBP panel to confirm the initial TBP panel result. Of the 16 samples, seven samples had dual-positive results by the TBP panel or were positive for a second pathogen not originally detected by LDT. These samples were tested by the LDT for B. microti, *E. chaffeensis*, or A. phagocytophilum. Of the 7 samples tested for dual positivity, 2 samples (TBP_144 and TBP_179) were dual positive for B. microti and A. phagocytophilum, and one sample (TBP_032) was dual positive for B. microti and *E. chaffeensis*, which confirmed these codetections.

**TABLE 2:** Discrepant analysis of dual-positive samples by the TBP assay

Sample ID^*a*^	ARUP result	Initial TBP result	Repeat TBP result	Additional ARUP LDT result	PCR and bidirectional sequencing	Final result^*b*^
TBP_144	A. phagocytophilum	A. phagocytophilum and B. microti	A. phagocytophilum and B. microti	B. microti *detected*	Not tested	TP A. phagocytophilum, TP B. microti
TBP_179	A. phagocytophilum	A. phagocytophilum and B. microti	A. phagocytophilum and B. microti	B. microti *detected*	Not tested	TP A. phagocytophilum, TP B. microti
TBP_032	B. microti	B. microti and *E. chaffeensis*	B. microti and *E. chaffeensis*	*E. chaffeensis detected*	Not tested	TP B. microti, TP *E. chaffeensis*
TBP_226	B. microti	B. microti and *E. chaffeensis*	*Borrelia* group 2	*E. chaffeensis* not detected	*E. chaffeensis* not detected, *Borrelia* group 2 not detected	TP B. microti, FP *E. chaffeensis*
TBP_358	A. phagocytophilum	A. phagocytophilum and *Borrelia* group 2	A. phagocytophilum and *Borrelia* group 2	*Borrelia* group 2 not detected	*Borrelia* group 2 not detected	TP A. phagocytophilum, FP *Borrelia* group 2
TBP_202	B. microti	B. microti and A. phagocytophilum	B. microti	A. phagocytophilum not detected	A. phagocytophilum detected	TP B. microti, A. phagocytophilum unresolved
TBP_367	*E. chaffeensis*	*E. chaffeensis* and B. microti	*E. chaffeensis* and *Borrelia* group 2	B. microti not detected	B. microti detected, *Borrelia* group 2 not detected	TP *E. chaffeensis*, B. microti unresolved

a ID, identifier.

b TP, true positive; TN, true negative; FP, false positive; FN, false negative.

The remaining four dual-positive samples by the TBP panel (TBP_226, TBP_358, TBP_202, and TBP_367) were tested by PCR and bidirectional sequencing. Samples TBP_226 and TBP_358 were determined to be false positive for the second target, *E. chaffeensis* and *Borrelia* group 2, respectively, based on negative codetections by PCR and lack of amplification with bidirectional sequencing. However, samples TBP_202, positive for B. microti and A. phagocytophilum, and TBP_367, positive for *E. chaffeensis* and B. microti, were unresolved, as repeat TBP panel testing and LDT were negative for the second target, but bidirectional sequencing was positive.

Discrepant analysis of the remaining nine samples was performed by repeat testing on the TBP panel or with bidirectional sequence analysis alone (Table 3). Four samples were false negative for B. microti, and in all four, either *Borrelia* group 1 or *Borrelia* group 2 was detected in the initial TBP test. Two of these discrepant samples (TBP_029 and TBP_043) repeat tested as B. microti by the TBP panel, while the other two (TBP_218 and TBP_264) were negative for B. microti both by the TBP panel and bidirectional sequencing. These two samples were low positives for B. microti by LDT, suggesting differences in the limits of detection between the LDT and TBP panel.

**TABLE 3:** Discrepant analysis for samples with incorrect amplification and step down

Sample ID	ARUP result	Original TBP result	Repeat TBP result	PCR and bidirectional sequencing	Final result^*a*^
TBP_176	A. phagocytophilum	No detection	No detection	No detection	FN A. phagocytophilum
TBP_029	B. microti	*Borrelia* group 2	B. microti	None	FN B. microti, FP *Borrelia* group 2
TBP_043	B. microti	B. microti and *Borrelia* group 1	B. microti	*Borrelia* group 1 not detected	TP B. microti, FP *Borrelia* group 1
TBP_218	B. microti	*Borrelia* group 2	Negative	B. microti not detected, *Borrelia* group 2 not detected	FP *Borrelia* group 2, FN B. microti
TBP_264	B. microti	*Borrelia* group 2	Negative	B. microti not detected, *Borrelia* group 2 not detected	FP *Borrelia* group 2, FN B. microti
TBP_205	*E. chaffeensis*	*Borrelia* group 1	*E. chaffeensis*	None	FP *Borrelia* group 1, FN *E. chaffeensis*
TBP_363	*E. chaffeensis*	No detection	*E. chaffeensis*	None	FN *E. chaffeensis*
TBP_193	*E. ewingii/E. canis*	No detection	No detection	*E. ewingii* not detected, *Ehrlichia* spp. not detected	FN *E. ewingii*
TBP_059	Negative	A. phagocytophilum	No detection	None	FP A. phagocytophilum

a TP, true positive; TN, true negative; FP, false positive; FN, false negative.

In the initial analyses, samples TBP_205 and TBP_363 were false negative for *E. chaffeensis*, with TBP_205 testing false positive for *Borrelia* group 1. Both samples tested as *E. chaffeensis* upon TBP panel repeat testing, suggesting PCR inhibition in the initial TBP panel run and/or incorrect assembly of the signal in channel 1 by the data analysis software. Sample TBP_193, which was positive for *E. ewingii*/*E. canis* by LDT, was not detected in the TBP assay nor by bidirectional sequencing. This suggested that the assay design is specific to *E. ewingii* and does not detect *E. canis* as demonstrated by the manufacturer in their exclusivity studies (14). Sample TBP_059 was determined to be a false positive for A. phagocytophilum on the initial TBP panel run and was not detected upon repeat testing. Last, sample TBP_176 was false negative for A. phagocytophilum and could not be resolved by repeat testing on the LDT or further analyzed due to sample depletion.

Following discrepant analyses and resolution, the PPA and NPA for the TBP panel were 97.7% (95% CI, 95.2% to 99.0%) and 99.6% (95% CI, 99.3% to 99.8%), respectively, compared to LDTs, with an overall agreement of 99.5% (95% CI, 99.2% to 99.7%).

## DISCUSSION

In this study, we evaluated the performance of a novel HDPCR TBP panel for the detection of tick-borne pathogens in whole-blood and synovial fluid specimens. Our results show that the TBP panel shows good concordance with validated LDTs and is capable of simultaneous detection of common tick-borne pathogens in a single-well, multiplex panel. The scalable throughput of the system allows for testing of up to 92 samples in less than 3 h. Moreover, the user-friendly cloud-based ChromaCode software allows for an easy and rapid analysis of the results efficiently within 2 to 3 min. The HDPCR technology can be readily adopted on other standard qPCR instruments, enhancing their ability to multiplex with 4 to 6 channels. Our evaluation was performed using the 96-well fast block on the QuantStudio 12K system, while other groups have evaluated this assay on the ABI 7500 FastDx instrument (Thermo Fisher Scientific, Waltham, MA) (11), highlighting the ease of adopting this assay on existing qPCR platforms.

The discrepancies in the results between the TBP panel and LDTs may be attributed to sample degradation of the frozen whole-blood samples, well-to-well contamination, differences in the assay limit of detection, variant sequences of the targets being amplified, or to the inclusivity of strains used in the TBP panel design. Additionally, incorrect assembly of the signal amplification curves or weak signal amplification due to low-positive samples, PCR inhibition, or probe mismatch can create false-negative and false-positive results. In our study, we observed the majority of false positives with B. microti-positive samples that tested incorrectly either as *Borrelia* group 1 (*n* = 2) or *Borrelia* group 2 (*n* = 3). These samples likely were low-positive B. microti or samples with a PCR inhibition resulting in a lower amplification signal intensity level, thus classifying the PCR curves incorrectly with the software, creating a false-positive *Borrelia* group 1 or group 2 result. This is a potential issue in the diagnosis of B. microti, especially in patients with mild infection/low-level parasitemia, for whom additional testing may be required. Further evaluation of the TBP panel B. microti target and software analysis algorithm may be warranted for improved accuracy and specificity. Furthermore, the *Babesia* target in the TBP panel is inclusive to the B. microti species and does not cross-react with other species that cause human infections, including *B. duncani* in the western regions of the United States, *B. divergens*, and unnamed strains designated MO-1 and EU-1. Fourteen samples positive for *Babesia* species other than B. microti by LDT tested negative by the TBP panel (data not shown). Though B. microti is the most common species in the United States, the TBP panel will miss these less-common *Babesia* spp., and diagnosis by microscopic examination of blood smears will still be necessary. The clinical implications of the false-positive or false-negative results are important to consider. Treatment with doxycycline or tetracycline as first-line treatment is recommended for Lyme disease, ehrlichiosis, anaplasmosis, tick-borne relapsing fever, and Rocky Mountain spotted fever. Though a false-positive Lyme *Borrelia* result for an *E. chaffeensis* infection highlights the analytical discrepancies of the assay, it may not result in a change in treatment or have a low impact on clinical care. In contrast, a false-positive relapsing fever *Borrelia* result for a B. microti infection may have severe implications, as B. microti requires treatment with atovaquone plus azithromycin or with clindamycin plus quinine. This limitation of the analytical performance could result in missed diagnoses and lack of appropriate directed therapy for babesiosis.

Our study has several limitations. First, with a retrospective study design, we tested deidentified samples known to be previously positive for *Ehrlichia* spp., A. phagocytophilum, B. microti, or Lyme *Borrelia* spp. in a reference laboratory. The positivity rates of these targets are higher than what may be observed in a prospective study due to a sampling bias. This study set was enriched for these positive specimens to better evaluate the analytical performance of the assay. Since the detection of Lyme *Borrelia* DNA in blood is exceedingly rare and has limited diagnostic utility (15), we included a limited number of positive synovial specimens to evaluate the analytical performance of the *Borrelia* group 1 (Lyme *Borrelia*) target in the TBP panel. Second, the whole-blood and synovial fluid specimens underwent at least 1 to 2 freeze/thaw cycles before extraction and TBP testing, which could result in false negatives due to sample degradation. Third, no cases of B. miyamotoi, relapsing fever *Borrelia* spp. (B. hermsii, *B. parkeri*, and *B. turicatae*), and *Rickettsia* spp. were identified in our study, limiting the evaluation of these targets.

Despite these limitations, our study is able to provide useful preliminary data on the analytical performance of this novel multiplex tick-borne panel using clinical specimens at a reference laboratory. Overall, the TBP panel assay is a novel, user-friendly method for the detection of common tick-borne pathogens in clinical specimens. This assay when used in areas of high incidence of tick-borne illnesses could impact the early detection of tick-borne pathogens and the early administration of treatment, which may contribute to better outcomes.
